# Perception of body translation amplitude in altered gravity during parabolic flight

**DOI:** 10.3389/fphys.2025.1595836

**Published:** 2025-05-21

**Authors:** Gilles Clément, Olga Kuldavletova, Gaëlle Quarck, Timothy R. Macaulay, Scott J. Wood, Pierre Denise

**Affiliations:** ^1^ KBR, Houston, TX, United States; ^2^ Université de Caen Normandie, INSERM, COMETE U1075, CYCERON, CHU de Caen, Caen, France; ^3^ NASA Johnson Space Center, Houston, TX, United States

**Keywords:** translation perception, time perception, vestibular system, altered gravity, space exploration

## Abstract

**Introduction:**

This study aimed to assess how individuals perceive the amplitude of passive body translation in microgravity and hypergravity.

**Methods:**

Six subjects participated in parabolic flights aboard the Novespace A-310 Zero-G aircraft, performing tasks that involved linear translation ranging from 25 to 250 cm across different axes, all while blindfolded. After each motion stimulus, subjects reported their perceived displacement, while trial duration and movement amplitude and dynamics were recorded.

**Results:**

Results showed that the perceived amplitudes of translations were accurate in 1 g. However, subjects significantly underestimated distances in 0 g and overestimated them in 1.8 g.

**Discussion:**

These findings suggest that, in microgravity, the lack of gravitational cues disrupts the vestibular system’s ability to provide accurate information on body movement, leading to altered motion perception. The role of temporal cues in estimating movement, particularly when gravitational input is altered, is inferred since the reports were made following each trial. Countermeasures such as visual aids and proprioceptive devices could help astronauts improve distance and time estimates during long-duration missions, especially in vehicles with restricted visibility or when operating rovers on Lunar or Martian terrains.

## Introduction

Passive self-motion is an integral part of everyday life, experienced while traveling by car, train, or airplane. The peripheral vestibular system detects head movement across six degrees of freedom using five specialized sensory organs. The three semicircular canals, oriented nearly perpendicular to one another, detect angular accelerations and transmit an angular velocity signal to the brain. This occurs through fluid-cupula dynamics that mechanically integrate acceleration into velocity, with the canal response varying by stimulation frequency—being more sensitive to higher-frequency head movements. The two otolith organs—the saccule and utricle—detect linear accelerations, including gravity, in both the horizontal and vertical planes (Goldberg and Fernández, 1975).

The perception of passive self-motion can be estimated by determining the minimum rotation or translation required for awareness, as well as by a subject’s ability to estimate displacement. Vestibular perceptual thresholds for translations along the X (naso-occipital) and Y (interaural) axes (1–2 cm/s^2^) are lower than those along the Z (longitudinal) axis (3–5 cm/s^2^). These values can vary depending on individual sensitivity, experimental conditions, and the influence of visual or somatosensory cues (Britton and Arshad, 2019).

Research conducted in aerobatics and parabolic flights shows that exposure hypergravity or hypogravity impairs the perception of self-motion. On Earth, when our body is passively tilted relative to gravity, the semicircular canals detect the angular velocity of the head tilt, while the otolith organs sense the change in the head’s perceived orientation with respect to gravity. During hypergravity conditions, such as high-G turns in aviation, individuals tend to overestimate their body tilt, a misperception known as the G-excess illusion (Lackner and DiZio, 2009; Stott, 2013). In contrast, when body tilt occurs in hypogravity environments, like lunar gravity experienced in parabolic flight, individuals tend to underestimate their body tilt (Meskers et al., 2021). Clark and Young (2017) coined the term “G-shortage” illusion to describe the underestimation of roll tilt in hypogravity. Changes in one’s ability to accurately perceive tilt (rotation) can influence the accurate perception of translation (Merfeld, 2003).

Studies conducted during spaceflight demonstrate that extended exposure to microgravity (0 g) causes a temporary deconditioning of the otolith system, which gradually recovers once back in Earth’s gravitational environment. In the only study on perceived translation during spaceflight, Arrott et al. (1990) found no significant changes in sensitivity to linear acceleration along the three axes in four subjects who were passively translated on a sled. However, during *active* body translation in both hypergravity and hypogravity conditions in parabolic flight, individuals experienced altered perceptions of distance: in hypergravity, subjects tended to overestimate the distance compared to normal gravity, while in hypogravity, they generally underestimated the magnitude of active body translation (Clément et al., 2016; 2020).

Previous studies conducted in parabolic flight have demonstrated that in microgravity, some subjects struggle to perceive the direction of motion, though they can still sense motion (Lackner and Graybiel, 1979). Therefore, in this study, instead of measuring vestibular perceptual thresholds—where subjects are asked to identify the direction of motion at the lowest possible acceleration—we focused on recording their perceived amplitude of motion.

Before this study, self-perception of *passive* body translation amplitude had not been assessed in microgravity. In this environment, the usual tonic stimulation of the otolith organs is absent due to weightlessness. Consequently, during whole-body translation in 0 g without visual input, individuals depend only on the inertial linear acceleration signals from the otoliths in the direction of motion, without the usual combination with gravitational acceleration. To estimate body movement, the central nervous system must integrate these linear acceleration cues from the otoliths, as well as somatosensory information. The objective of this study was to assess the accuracy of self-perception of passive translation along the X, Y, and Z-axes in microgravity and hypergravity.

## Methods

### Participants

Six subjects (four females, two males; average age 36.7 ± 9.9 years) participated in one parabolic flight campaign in 2024 (85th ESA campaign) aboard the Novespace A-310 Zero-G aircraft in Bordeaux, France. The campaign consisted of three flights, with 31 parabolas per flight. Each parabola lasted approximately 25 s in microgravity (0 g), with the pull-up and pull-out phases (at 1.8 g) occurring before and after each parabola, each lasting 20 s. Subjects were tested during 15 parabolas in each campaign.

Five of the six subjects had prior experience with parabolic flight. To prevent motion sickness, all participants opted for a prophylactic use of scopolamine (0.075–0.235 mg). One experienced subject had motion sickness during Y-axis translations but continued the test. Since scopolamine acts as a vestibular suppressant and may affect task performance, we ensured that responses at 1 g during flight (with medication) were consistent with those at 1 g on the ground without medication. Because subjects were medicated at all gravity levels (1 g, 0 g, and 1.8 g) during the flight, any medication-related effects were accounted for through repeated-measures statistical analysis.

The test procedures were approved by a French Ethical Committee (*Comité de Protection des Personnes de la Région Ouest Ile de France VIII,* n°ID-RCB 2024-A01524-43) and were conducted in accordance with the ethical standards outlined in the 1964 Declaration of Helsinki. All subjects provided written informed consent prior to participating in the study.

### Experimental protocol

**FIGURE 1:**
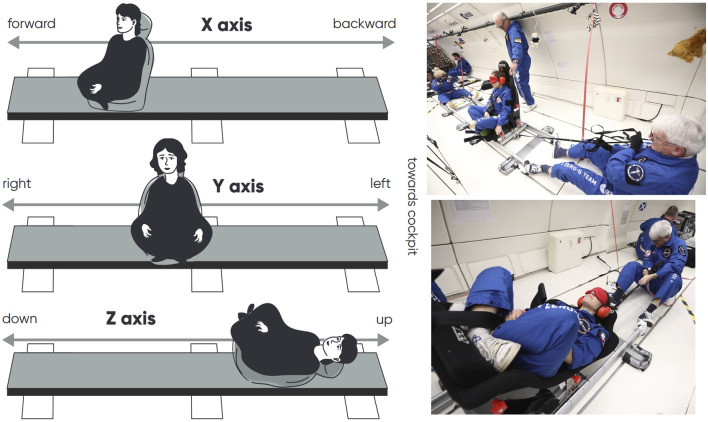
Left: Drawing of the method used to evaluate the perception of translation along the X, Y, and Z-axes. Right: Photographs of translation trials along the Y and Z-axes. Photo Credit Nicolas Courtioux, Novespace.

The perception of body translation amplitude was evaluated using a 3-m linear sled, with subjects sitting along the direction of motion, sitting sideways, or lying on their back (Figure 1). Subjects were translated along the fore-aft (X-axis), left-right (Y-axis), or up-down (Z-axis), with the direction and amplitude of movement randomly varying between 25 cm and 250 cm. During translations along the X, Y, and Z-axes, subjects had their back, left side, or head facing the airplane’s cockpit, respectively. Two operators controlled the sled, with one pulling in one direction and the other in the opposite direction. Each trial began with the sled positioned at the same endpoint on the rail.

Operators were trained to pull the sled with consistent force to maintain a relatively constant velocity across trials. However, the forward acceleration of the airplane during the pull-up phase (∼0.2 g) made it more difficult for the operator facing away from the cockpit to move the subjects in the forward direction (X-axis), to their left side (Y-axis), and upward (Z-axis). Despite this, the linear acceleration of the sled remained significantly above the vestibular thresholds for detecting linear accelerations (Gianna et al., 1996).

After each trial, a third operator recorded the precise sled position using markings on the sled tracks and the perceived translation amplitude (in centimeters) as reported by the subject. Trials were conducted under the following conditions: (a) in 1 g on the ground while the aircraft was stationary on the runway, (b) in 1 g during straight and level flight aboard the aircraft between parabolas, (c) during the 1.8 g pull-up phase, and (d) during the 0 g phase. Three different amplitudes were tested during each parabola, with subjects providing distance estimates after each translation.

The subjects were unaware of the upcoming displacement. Operators were provided with 9 target amplitudes for each parabola (3 target amplitudes per gravity level), generated by an algorithm that created a random sequence of 270 target amplitudes using 30-cm steps for each subject (90 target amplitudes per axis). The algorithm ensured alternating sled motion directions and prevented any amplitude from being repeated more than three times. Once the sled stopped, it was not adjusted to perfectly match the target amplitude. The measured error between the target and actual motion amplitudes was below 10%, enabling data collection across a relatively continuous range of amplitudes rather than at discrete, fixed values.

The subjects wore a blindfold and external noise-cancelling earphones to eliminate any visual and auditory cues that could provide information about spatial orientation. The subjects were secured in a padded racing car seat using a 5-point harness, which restricted body movements, while the seat’s headrest further limited head motion in the roll and yaw axes.

Training sessions before each flight ensured that both subjects and operators were well-prepared for the tasks. The training sessions consists of 9 motion amplitudes, similar to those performed during a single parabola trial. Video recordings of every trial across all tests were captured using GoPro cameras securely mounted on the aircraft rails. These recordings were later analyzed for measuring the duration of each trial. The mean sled velocity was calculated by dividing the actual sled amplitude by the duration of each trial. Triaxial inertial measurement units (WitMotion, Shenzhen, China) were securely mounted to the subject’s seat to record sled motion. Unfortunately, the acceleration data were excluded from analysis due to high-frequency vibration interference from the aircraft turbulence and an insufficient sampling frequency to accurately capture the sled’s motion dynamics.

### Statistical analysis

The differences (errors) between the subjects’ judgments and the actual amplitudes of translation were calculated for each trial. As noted earlier, subjects experienced slightly different translation amplitudes; therefore, to normalize their responses, we calculated the ratio of perceived distance to actual distance, referred to as perception gain. Our primary hypothesis was that the perception of motion amplitude—the relationship between actual and perceived distance—was influenced by the gravity level. Additionally, the mean sled velocity for each trial was calculated as the ratio of actual distance to trial duration.

Welch’s t-test was used to evaluate differences in translation perception gains between 1 g on the ground and 1 g in flight along the X, Y, and Z-axes. P-values were adjusted for multiple comparisons using Holm’s method.

A robust linear mixed-effects model was used to assess the influence of gravity and potential confounding variables on translation perception gain. This model was chosen because the residuals of a standard linear mixed-effects model were not normally distributed. The following model was fitted:

$$\mathrm{perception}\;\mathrm{gain}\;∼\mathrm{mean}\;\mathrm{sled}\;\mathrm{velocity}+\mathrm{gravity}\;*\;\mathrm{axis}+\mathrm{motion}\;\mathrm{direction}\;\mathrm{relative}\;\mathrm{to}\;\mathrm{the}\;\mathrm{cockpit}+\left(1|\mathrm{subject}\right)$$

This formulation allowed for the evaluation of the effects of gravity level and translation axis, as well as their interaction, while controlling for the direction of motion relative to the cockpit and mean sled velocity. Subject-level variability was modeled as a random effect. The analysis was conducted in **R** (R Core Team, 2022) using the rlmer function from the **robustlmm** package, which reduces the influence of outliers on both fixed and random effects. Approximate *p*-values were obtained using the **sjPlot** package, which derives significance estimates based on the degrees of freedom from a corresponding non-robust mixed-effects model.

## Results

**FIGURE 2:**
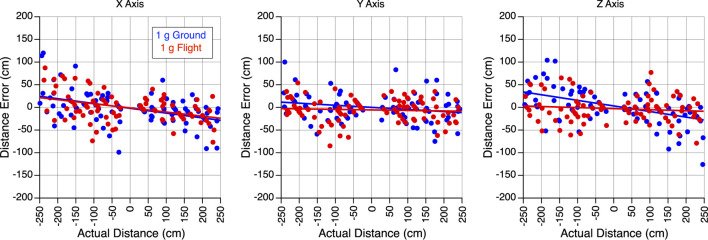
Perception of translation in 1 g. Error in perceived distance during body translations along the X, Y, and Z-axes in 1 g on the ground and 1 g during the flight. Positive distances correspond to the forward, rightward, and downward directions, respectively.

After applying Holm’s correction for multiple comparisons, Welch’s t-test revealed no significant differences in perception gains (i.e., the ratio between perceived translation distance and actual translation distance) during body translations along the X-axis (t = 0.341, df = 167.12, *p* = 0.734), the Y-axis (t = 0.904, df = 182.04, *p* = 0.734) or the Z-axis (t = 1.982, df = 171.79, *p* = 0.147) between 1 g on the ground and 1 g during flight (Figure 2).

**FIGURE 3:**
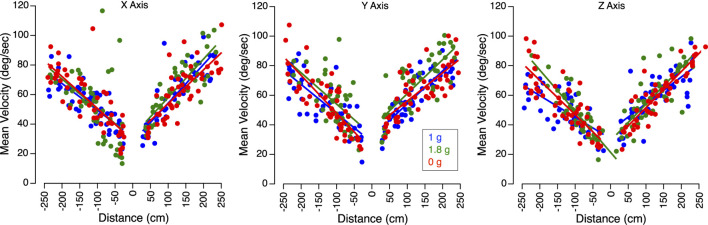
Velocity of translation trials. Mean sled velocity for X, Y, and Z-axes translations in 1 g, 1.8 g, and 0 g during the flight. Positive distances correspond to the forward, rightward, and downward directions, respectively.

**FIGURE 4:**
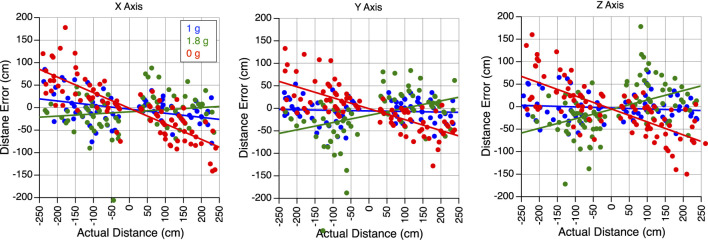
Perception of translation in altered gravity. Error in perceived distance during body translations along the X, Y, and Z-axes in 1 g, 1.8 g, and 0 g during the flight. A negative slope signifies an underestimation of distance, while a positive slope denotes an overestimation of distance.

In all gravity conditions, mean sled velocity increased with the amplitude of body motion for translations along the X, Y, and Z-axes (Figure 3). Perceived translation distances were generally accurate in 1 g, significantly underestimated in 0 g, and overestimated in 1.8 g (Figure 4).

**FIGURE 5:**
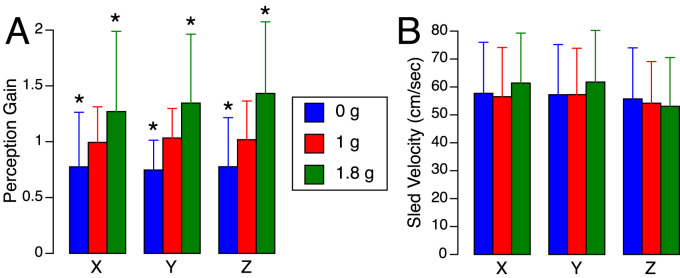
Translation perception gains **(A)** and mean sled velocities **(B)** averaged for each body axis and gravity level during the flight. Mean ± standard deviation of all trials in six subjects. *p < 0.05 relative to 1 g.

Figure 5 presents the perception gains and mean sled velocities averaged across all subjects and trials. A two-factor repeated-measures ANOVA (gravity level, axis) showed no significant differences in the perception gain across subjects (F (5,53) = 0.867, *p* = 0.512). The robust linear mixed-effects model revealed a significant effect of gravity level on perception gain, with no significant differences observed across translation axes. However, perception gain was significantly influenced by both sled velocity and direction of motion: slower motions were associated with larger distance errors. As seen in Figure 3, sled movements were slower during displacements toward the cockpit—that is, when subjects moved backward, leftward, or upward. As described in the Methods section, these asymmetries in mean sled velocity under the 1.8 g condition were likely due to operational constraints. Specifically, sled movements toward the cockpit were more difficult to execute during the airplane’s pull-up phase, when forward acceleration made precise control more challenging (Table 1).

**TABLE 1:** Results of the mixed linear model for translation perception gain.

Fixed effects	Perception gain			
Predictors	Estimates	Confidence interval	t-value	p-value
(intercept)	1.29	1.15 to 1.43	18.09	<0.001*
Gravity [0 g] (difference between 1 g and 0 g)	−0.24	−0.33 to −0.15	−5.16	<0.001*
Gravity [1.8 g] (difference between 1 g and 1.8 g)	0.21	0.12 to 0.30	4.48	<0.001*
Axis [Y] (difference between X and Y-axes)	0.06	−0.03 to 0.15	1.39	0.167
Axis [Z] (difference between X and Z-axes)	0.03	−0.06 to 0.12	0.71	0.317
Effect of sled velocity	−0.01	−0.01 to 0.00	−8.90	<0.001*
Effect of motion direction	0.05	0.00 to 0.09	2.06	0.040*
Interactions Gravity [0 g] x-Axis [Y] (difference between X and Y-axes in 0 g)	−0.03	−0.16 to 0.10	−0.49	0.623
Gravity [0 g] x-Axis [Y] (difference between X and Y-axes in 0 g)	0.05	−0.08 to 0.18	0.73	0.467
Gravity [1.8 g] x-Axis [Y] (difference between X and Y-axes in 1.8 g)	−0.01	−0.14 to 0.12	−0.12	0.904
Gravity [0 g] x-Axis [Z] (difference between X and Z-axes in 0 g)	0.12	−0.01 to 0.25	1.74	0.082

Random Effects	Variance	Standard deviation
Subject (intercept)	0.01287	0.1134
Residual	0.09303	0.3050
Number of observations: 812		
Groups: subject, 6		

The model included, gravity level, translation axis, mean sled velocity, and direction of motion as fixed factors, and subject as a random factor. The direction of motion toward the cockpit was as follows: backward (X-axis), to the left (Y-axis), and upward (Z-axis). For fixed effects, the robust estimation down-weighted 22% of the residuals. None of the random effects were down-weighted. *p < 0.05.

In 0 g, during translation along the Z-axis (moving up or down), one subject reported the sensation of riding over a hill in the middle of the track, a phenomenon known as the hilltop illusion (Young, 1984). The perceived height of the hill was 50 cm. During translation along the X-axis (moving forward and backward), the illusion was also present, though with a smaller perceived height of 20 cm. Notably, the hilltop illusion did not occur during translation along the Y-axis.

## Discussion

Although vestibular input plays a dominant role in translation motion perception (Kobel et al., 2024), self-motion perception relies on the integration of multiple sensory inputs, including visual and proprioceptive. When these sensory signals provide conflicting information the brain’s processing of movement can be altered, leading to distorted perception, disorientation, or motion sickness. The perception of linear motion in 0 g is significantly altered due to the absence of a stable gravitational reference which disrupts how the brain interprets signals from the vestibular system. Our findings indicate that translation amplitude is systematically underestimated in 0 g. In 1 g, the otolith organs detect both gravitational and inertial accelerations, allowing the brain to distinguish between gravity and self-motion. In 0 g, however, the otoliths respond solely to inertial acceleration, leading to errors in motion perception (Young et al., 1984). Conversely, in hypergravity conditions–such as the 1.8 g pull-up phase of a parabolic flight–translation amplitude is overestimated. This suggests that the brain relies on an expected ratio between inertial and gravitational forces. When gravity increases, acceleration is perceived as stronger, resulting in an overestimation of motion (Lackner and DiZio, 2000).

However, both hypergravity and microgravity significantly alter proprioceptive input as well. Vestibular information is integrated with proprioceptive and visual inputs beginning at the level of the second-order vestibular neurons (Cullen, 2012). As a result, it is the processing of all sensory inputs—not just otolith signals—that is affected by altered gravity. One could speculate that, due to the convergence of proprioceptive and otolith inputs onto second-order vestibular neurons, a reduction in proprioceptive signals in microgravity may lead to decreased excitation of these neurons, while increased proprioceptive input in hypergravity may cause greater depolarization.

In a previous study conducted in parabolic flight, subjects reproduced distances while actively translating on a sled (Clément et al., 2016; 2020). The findings revealed that subjects stopped short of the target distance when translating along the X-axis in 1.8 g compared to 1 g. In distance reproduction tasks, stopping short suggests that subjects overestimated the distance as they perceived they had traveled the full distance even though they had not. Conversely, subjects traveled farther than the target distance in 0 g compared to 1 g when translating along the Z-axis, indicating an underestimation. This overestimation of distance in 1.8 g and underestimation in 0 g compared to 1 g are consistent with the results of the present study.

Other studies have demonstrated that translation perception can vary with motions along different body axes and body orientations (Diaz-Artiles and Karmali, 2021). One limitation of the present study is that the body orientation relative to gravity (relevant for 1 g and 1.8 g measures) for Z-axis was maintained in a horizontal plane due to the constraints of the aircraft while the body orientation was upright for X and Y-axes. While we did not observe changes between X, Y, and Z-axes across any gravity level, based on prior research one might expect a reduction in accuracy in the Z-axis during Earth vertical movements (Jones and Young, 1978). Another limitation of this study is that the detailed acceleration profiles were not measured, although they constituted the actual stimuli delivered to the vestibular system. As such, any irregularities–such as abrupt changes or subthreshold segments–may have introduced potential confounds in the vestibular system’s double integration process.

Our results from parabolic flights differ from those observed in spaceflight. Arrott et al. (1990) reported no significant changes in sensitivity to linear acceleration along the three axes in four subjects passively translated on a sled during spaceflight, although they did report increased inter-subject variability. This same study showed enhanced performance on a closed-loop nulling tasks early postflight, suggesting increased perceptual gains. Consistent with this, other studies have shown that perceived translation amplitude increased in eleven subjects shortly after spaceflight during sinusoidal oscillations on a sled and during off-vertical axis rotation (Clément and Wood, 2013; 2014). The study by Arrott et al. (1990) was conducted after 1 day in space; therefore, another possible explanation for the discrepancy is that their subjects had already begun adapting by recalibrating the relative weighting of vestibular, visual, and proprioceptive inputs, resulting in a more accurate perception of movement.

It has been proposed that the brain builds internal models based on previous sensory experiences. The internal model of gravity, informed by multimodal sensory cues, helps maintain balance and spatial orientation. However, when these gravity cues are absent as in microgravity, the brain’s internal model would become less effective at determining body movement (Lackner and Dizio, 2000; Merfeld, 2003). Some studies suggest that this internal model adapts over time during spaceflight, but the adaptation remains incomplete or occurs slowly (Glasauer and Mittelstaedt, 1998; Clément et al., 2001).

In our study, during the translation task, subjects waited until the stimulus was completed before estimating how far they had moved. Their judgments during translations were shaped by complex processing involving otolith and proprioceptive inputs, individual perceptual-motor styles, and varying sensitivity to scopolamine. Alternatively, the subjects may have also used trial duration as a reference for estimating distances. Instead of explicitly integrating vestibular and proprioceptive signals, subjects might estimate displacement by associating longer trial durations with greater movement and shorter durations with smaller movement. This strategy aligns with previous research on temporal perception in self-motion, which has shown that humans can infer distance based on the duration of motion when velocity is stable (Choi et al., 2021; Navarro Morales et al., 2025).

The reliance on time as a cue may be particularly relevant in microgravity, where the usual gravitational reference for motion perception is absent, making other cues like vestibular and proprioceptive signals less reliable. In such conditions, subjects may default to using more accessible temporal cues, consciously or unconsciously, to form their movement estimates. However, this strategy could introduce biases if variations in velocity are not accounted for, potentially leading to overestimations or underestimations depending on the perceived duration-motion relationship. Time perception has been shown to be altered in microgravity, both during parabolic and orbital spaceflight (Clément, 2018; Kuldavletova et al., 2023). This change in time perception could contribute to the altered distance estimates observed in 0 g in our study.

This study highlights challenges astronauts may face during missions to the Moon or Mars, where the vestibular system will not be adapted to hypogravity (Clément et al., 2022). Without reliable gravity cues, astronauts could struggle with spatial orientation, particularly when navigating in low-visibility conditions or operating vehicles. Inaccurate motion perception could impair tasks requiring precision, such as driving rovers or performing other tasks in exploration settings. To address these issues, countermeasures like visual aids (e.g., digital displays or augmented reality) that track distance and proprioceptive aids (e.g., haptic feedback or wearable devices) that provide orientation cues will be essential (Clément et al., 2018). These tools will help astronauts maintain spatial orientation, improving operational efficiency and safety during missions in low-gravity environments, until the brain adapts to conflicting sensory inputs through processes such as vestibular habituation and sensory adaptation.

## Data Availability

The raw data supporting the conclusions of this article will be made available by the authors, without undue reservation.
